# Selective demyelination of a sciatic nerve allograft after SARS-CoV-2 infection – Case report

**DOI:** 10.1016/j.heliyon.2023.e20624

**Published:** 2023-10-04

**Authors:** Magdalena Koszewicz, Dorota Kaminska, Jacek Martynkiewicz, Adam Domanasiewicz, Jerzy Gosk

**Affiliations:** a Department of Neurology, Wroclaw Medical University, Borowska 213, 50-556, Wroclaw, Poland; b Department of Nephrology and Transplantation Medicine, Wroclaw Medical University, Borowska 213, 50-556, Wroclaw, Poland; c Department of Trauma and Orthopedic Surgery, Regional Specialist Hospital, Kamienskiego 73a, 51-124, Wroclaw, Poland; d Department of Biomedical Engineering, Faculty of Fundamental Problems of Technology, Wroclaw University of Science and Technology, Grunwaldzki Sq. 13 (D-1), 50-377, Wroclaw, Poland

**Keywords:** Nerve injury, Nerve allograft, SARS-CoV-2, Demyelination, Intravenous immunoglobulins

## Abstract

Peripheral neurological complications are seen after SARS-CoV-2 infections. These are mostly immune-mediated such as Guillaine–Barré syndrome or chronic inflammatory demyelinating polyneuropathy. We present a 39-year-old man treated with a right sciatic nerve allotransplantation with subsequent clinical and electrophysiological improvement within 30 months of observation. After SARS-CoV-2 infection, he developed clinical deterioration with selective sciatic nerve demyelination in a nerve conduction study. Nerve conduction velocity returned to previous values within six months of treatment. Intravenous immunoglobulins were used at standard dosage. The inflammatory immune process seemed to be a cause of peripheral demyelination isolated to a nerve allograft with good reaction for intravenous immunoglobulin treatment.

## 1 Introduction

SARS-CoV-2 infection can cause a wide range of different complications, including neurological once: headache, taste and smell loss, dizziness, consciousness impairment; less commonly – cerebrovascular episodes, seizures, meningitis or encephalitis, and peripheral nervous system impairment. Peripheral neurological complications seem to be immune-mediated, and Guillaine–Barré syndrome has been described most often [[1], [2], [3]]. Complications following Covid-19 infection or vaccination have also been described in transplant patients. Well documented rejection of solid organs has been reported in a small group of patients, mainly after cornea and kidney transplantation [4]. In recent, years the surgical techniques, and medical technologies of nerve allograft engineering have developed, resulting in improved final treatment results, and decreased immunological response, but the need for immunosuppressive treatment remains [5,6]. In the available literature nerve rejection connected with COVID-19 infection has not been described. We have also not found a case of a demyelinating process limited to the area of the transplanted nerve without concomitant systemic features indicating graft rejection in the course of SARS-CoV-2 infection or as a complication of vaccination.

### 1.1 Clinical story

The authors have received a the patient's written consent form.

**Fig. 1:**
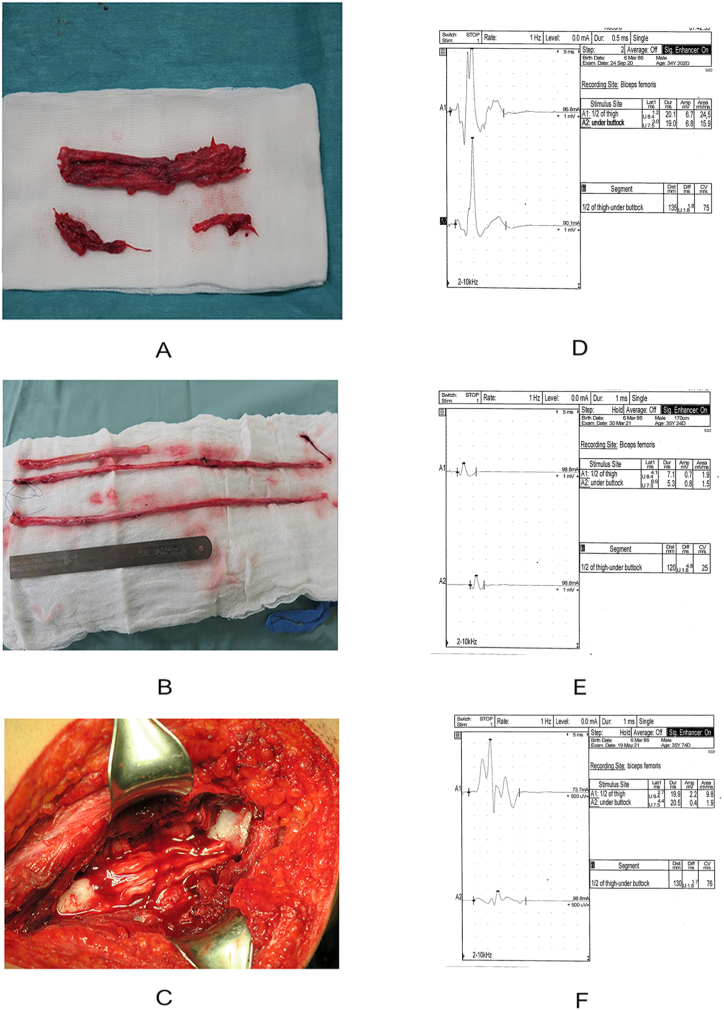
Excised damaged sciatic nerve after inguinal gunshot (A). Procured upper limb nerve allografts – 16cm in length, consisting of the donor's radial, median and ulnar nerves (B), and transplanted allografts to the sciatic nerve defect (C). Right sciatic nerve conduction studies: recording needle electrode, recording site: *biceps femoris*, stimulation sites: mid-point of the thigh, and under the buttock. 26 months (D) after allotransplantation – parameters were within normal limits; 2 months after Covid-19 infection (E) – severe slowing of conduction velocity to 25 m/s; and after 6 months of IgIV treatment (F) – conduction velocity returned to normal limits (76 m/s).

A 39-year-old healthy man, police officer, was treated with right sciatic nerve allotransplantation after being shot. A multifragmental fracture of the femur neck and an initially 12 cm defect of the sciatic nerve were observed (Fig. 1A). The 16 cm nerve allograft consisted of the donor's radial, median and ulnar nerves (Fig. 1B and C) is described elsewhere. The patient received triple immunosuppresion (tacrolimus, mycophenolate mofetil, prednisone) with induction of basiliximab. Mycophenolate mofetil and predisnode were gradually tapered and finally stopped after two years. Tacrolimus was stopped after three years. Within 30 months of the nerve transplantation we observed a slow, gradual recovery of the function of the posterior thigh muscles, without any improvement of the peroneal and tibial nerve functions. On the Lovett scale the gradual improvement ranged from one point at baseline to a value three/four points after 30 months. After cessation of immunosuppression the patient suffered from COVID-19 infection. Within 2 months of SARS-CoV-2 infection, the patient again reported progressive weakness of the proximal muscles of the right lower limb. In the neurological examination the muscle strength was rated as 2 on the Lovett scale. Neither, general weakness, nor sensory disturbances were observed. Intravenous immunoglobulins (IVIg) at standard dosage were used every six weeks [3]. Within six months of treatment, a significant improvement in posterior tight muscle strength was observed, which returned to the state before COVID-19 infection, and it was rated at 3/4 on the Lovett scale. We did not observe any adverse events.

### 1.2 Nerve conduction studies

After the allotransplantation nerve conduction studies were performed several times. In repeated neurographic examinations, no recovery of peroneal and tibial nerve function was observed, which was consistent with the clinical picture. A non-standard nerve conducting study was performed in order to assess the function of the sciatic nerve, however the method was based on data from the literature [7,8]. The stimulation sites were in the ½ of the tight (midpoint of the thigh), and under the buttock. The distance between the two points of stimulation varied between 12 and 14cm in the subsequent studies. We used monopolar stimulating electrode. The duration of the electrical stimulus was about maximal, and equal 0.7–1 ms. Motor potentials were collected from the biceps femoris muscle (short head) using a concentric needle electrode (50 × 0.45mm). The site of the needle electrode insertion was determined in a similar way for each examination, using anatomical landmarks - we measured 12 cm above the distal point on the line between ischial tuberosity and lateral post knee crease. We believe that the patient's own sciatic nerve was stimulated at the distal point of stimulation (1/2 of the tight), while the graft was stimulated at the proximal point (under the buttock). We were able to show progressive improvement of the sciatic nerve conduction velocity. An electrophysiological test performed 30 months after the allotransplantation showed normal values for sciatic nerve conduction in the middle upper part of the thigh (Fig. 1D). In the electrophysiological study performed after COVID-19 infection we revealed demyelination of the sciatic nerve in the middle upper part of the thigh with conduction velocity slowing to 25 m/s, prolongation of the distal latency (1.2 v 4.1 ms) and a lowering of the amplitude of motor potentials (Fig. 1E). There were no pathological findings in the electrophysiological tests of peripheral nerves in the left lower limb typical for demyelination, such as slowing of conduction velocity, distal latency prolongation or temporal dispersion. Within six months of treatment with IVIg the nerve conduction velocity in the right sciatic nerve returned to previous values (76 m/s) (Fig. 1F), i.e. those obtained in a study performed 30 months after the nerve transplant procedure. The amplitude of the potentials was still lower than previously, while the duration of the potentials was significantly longer, which might have corresponded to the temporal dispersion – probably a residual symptom of demyelination.

## 2 Discussion

Guillain–Barré syndrome (GBS), chronic inflammatory demyelinating polyneuropathy (CIDP) and CIDP variants such as multifocal acquired demyelinating sensory and motor neuropathy (previously MADSAM) have been described after SARS-CoV-2 infections [[1], [2], [3],^9^]. Isolated reports of mononeuropathy multiplex, and individual nerve damage are also present in the literature. The aetiology of these neuropathy remains poorly known, potential explanations include mechanical factors, vasculitis and endotheliopathy, the “cytokine storm”, and the molecular mimicry mainly in GBS associated with SARS-CoV-2 infection [[10], [11], [12]].

In the differential diagnosis GBS had to be considered in our patient. However he did not meet the time criterion for GBS according to the Brighton criteria [13]. Also, various studies have found that the mean duration between the onset of COVID-19 infection and GBS ranged from 11 to 25 days [12]. In the presented case, the period between COVID-19 infection and the development of clinical symptoms lasted about 2 months. Therefore, a diagnosis of CIDP seemed more likely. According to the European Task Force-Second Revision on CIDP [9] the patient seemed to meet the clinical criteria for focal variant of CIDP. Because the electrophysiological criteria were fulfilled in only one nerve, the diagnostic certainty could only be at a level of possible focal CIDP. CIDP variants probably have different mechanisms from typical CIDP, but they present features of demyelination and good response to immune therapy [9].

A limitation of the study may be the use of a needle electrode as a receiving electrode. Such a method may presentless reproducibility of results. The latency of CMAP depends on the needle position, the amplitude depends on the excitation of a group of fibres (not all of them), the shape of the potential is always multiphase. Nevertheless, it is an established technique for neurographic examination in difficult cases [7]. In our patient, it was the only research method possible immediately after the surgery. A later change from needle receiving electrodes to surface electrodes would make prevent a comparison of obtained results. Thanks to the method used, with the same procedures for each test, it was possible to monitor the patient.

Nerve allograft demyelination in our patient seemed to be provoked by SARS-CoV-2 infection. Immune-mediated inflammation is thought to be a possible cause of peripheral demyelination [1]. The demyelinating process was isolated to only one nerve which was probably more vulnerable than others – nerve allograft (*locus minoris resistentiae*). Intravenous immunoglobulins are thought to be the treatment of choice [3] in such cases and provide a good outcome; this was the case our patient.

## Author contribution statement

All authors listed have significantly contributed to the investigation, development and writing of this article.

## Data availability statement

Data included in article/supp. material/referenced in article.

## Funding sources for study

Supported by 10.13039/501100009687Wroclaw Medical University SUBZ.C220.23.073.

## Declaration of competing interest

The authors declare that they have no known competing financial interests or personal relationships that could have appeared to influence the work reported in this paper.
